# Time trends and social inequalities in child malnutrition: nationwide estimates from Brazil’s food and nutrition surveillance system, 2009–2017

**DOI:** 10.1017/S1368980021004882

**Published:** 2022-12

**Authors:** Rita de Cássia Ribeiro-Silva, Natanael de Jesus Silva, Mariana Santos Felisbino-Mendes, Ila Rocha Falcão, Rafaella da Costa Santin de Andrade, Sara Araújo Silva, Eduardo Augusto Fernandes Nilson, Ana Maria Spaniol, Rosemeire Leovigildo Fiaccone, Enny Paixão, Maria Yury Travassos Ichihara, Gustavo Velasquez-Melendez, Maurício Lima Barreto

**Affiliations:** 1 School of Nutrition, Federal University of Bahia, Av. Araújo Pinho, nº 32, Canela, CEP 40.110-150, Salvador, BA, Brazil; 2 Center for Data and Knowledge Integration for Health, Oswaldo Cruz Foundation, Salvador, BA, Brazil; 3 Department of Maternal and Child Nursing and Public Health, Nursing School, Federal University of Minas Gerais, Belo Horizonte, MG, Brazil; 4 General-Coordination Office for Food and Nutrition Policy, Ministry of Health, Brasília, DF, Brazil; 5 Institute of Mathematics and Statistics, Federal University of Bahia, Salvador, BA, Brazil; 6 Epidemiology and Population Health, London School of Hygiene and Tropical Medicine, London, UK; 7 Institute of Collective Health, Federal University of Bahia, Salvador, BA, Brazil

**Keywords:** Malnutrition, Infant, Preschool child, Time trends, Brazil

## Abstract

**Objective:**

In Brazil, national estimates of childhood malnutrition have not been updated since 2006. The use of health information systems is an important complementary data source for analysing time trends on health and nutrition. This study aimed to examine temporal trends and socio-demographic inequalities in the prevalence of malnutrition in children attending primary health care services between 2009 and 2017.

**Design:**

Time trends study based on data from Brazil’s Food and Nutrition Surveillance System. Malnutrition prevalence (stunting, wasting, overweight and double burden) was annually estimated by socio-demographic variables. Prais–Winsten regression models were used to analyse time trends. Annual percent change (APC) and 95 % CI were calculated.

**Setting:**

Primary health care services, Brazil.

**Participants:**

Children under 5 years old.

**Results:**

In total, 15,239,753 children were included. An increase in the prevalence of overweight (APC = 3·4 %; *P* = 0·015) and a decline in the prevalence of wasting (–6·2 %; *P* = 0·002) were observed. The prevalence of stunting (–3·2 %, *P* = 0·359) and double burden (–1·4 %, *P* = 0·630) had discrete and non-significant reductions. Despite the significant reduction in the prevalence of undernutrition among children in the most vulnerable subgroups (black, conditional cash transfer’s recipients and residents of poorest and less developed areas), high prevalence of stunting and wasting persist alongside a disproportionate increase in the prevalence of overweight in these groups.

**Conclusions:**

The observed pattern in stunting (high and persistent prevalence) and increase in overweight elucidate setbacks in advances already observed in previous periods and stresses the need for social and political strategies to address multiple forms of malnutrition.

One in three children worldwide presents impaired growth, as evidenced by at least one of the more visible malnutrition indicators (stunting, wasting, underweight or overweight/obesity)^(1)^. Estimates from the 2019 report by the United Nations Food and Agriculture Organization indicate that the world is not on track to meet global nutrition goals, including reducing the incidence of low birth weight and growth deficits among children up to 5 years of age^(2)^. In addition, rates of overweight and obesity continue to rise worldwide, especially among schoolchildren and adults^(2)^. In some children, short stature can occur simultaneously with overweight/obesity^(3)^. This phenomenon, sometimes referred to as the ‘double burden of malnutrition’ (DBM), has been commonly observed among the most impoverished populations^(4,5,6)^. It is associated with the rapid and inequitable nutritional transition process in progress in several low- and middle-income countries^(6)^. These multiple early manifestations of malnutrition represent risk factors for adverse health consequences throughout affected individuals’ lifetimes^(7,3)^.

Social, economic and political conditions are important structural determinants of malnutrition, which has increased among countries and in regions with exacerbated social inequality^(8,9,10,3)^. Despite making significant advances in reducing poverty and improving its population’s health and overall socio-economic conditions, Brazil ranks high among countries suffering from income inequality^(11)^. Unfortunately, this scenario has worsened in recent years, as evidenced by an increasing trend towards greater income inequality as measured by the Gini index since 2015. That happens in conjunction with severe austerity measures to reduce social policies’ financing aimed at reducing inequity^(12)^. In 2019, the country’s extremely impoverished population totalled 13·6 million, around 100 000 more people than the previous year. In 2014, when Brazil experienced lower unemployment rates, almost 5 million fewer people suffered from extreme poverty^(13)^. Recent data indicate that of the 68·9 million households in Brazil, 36·7 % (25·3 million) experience some degree of food insecurity, with the country’s poorest regions being more affected^(14)^.

Given this changing scenario, the assessment of trends in nutritional outcomes focused on specific population groups are extremely important, especially those greater vulnerability situations. In Brazil, up-to-date information nationally representative of childhood nutritional status has been non-existent since 2006 when the last National Demographic and Health Survey of Children and Women was performed. A substantial reduction in stunting prevalence (from 37 to 7 %) was observed between 1974 and 2006, as well as stability in the prevalence of overweight in children (from 6 to 7 %)^(15)^. Meanwhile, the Brazilian Ministry of Health operates the Food and Nutrition Surveillance System (SISVAN), which routinely monitors the food consumption and nutritional status of individuals receiving care at primary healthcare facilities throughout the national Unified Health System (SUS). SISVAN also incorporates anthropometric data from children whose health is monitored in accordance with the Bolsa Família conditional cash transfer program^(16)^. Between 2008 and 2017, SISVAN followed per year an average of 3·6 million children under 5 years of age^(17)^.

Given the severe setback in the Brazilian socio-economic scenario in recent years, together with the lack of recent available data regarding child malnutrition in Brazil, the present study aimed to examine temporal trends and social and regional inequalities in the prevalence of stunting, wasting, overweight/obesity and the double burden of malnutrition in children served by primary health care services in the Brazil’s Unified Health System between 2009 and 2017. These findings have the potential to show how the malnutrition burden falls disproportionately in the Brazilian population and assist in elaborating strategies that can target high-risk groups and contribute to the prevention and control of multiple forms of malnutrition, thereby supporting the achievement of the Sustainable Development Goals outlined in the United Nations 2030 Agenda, including efforts to eradicate hunger and malnutrition and to promote health and well-being, including the prevention of diet-related non-communicable diseases.

## Methods

### Study design and population

**Fig. 1:**
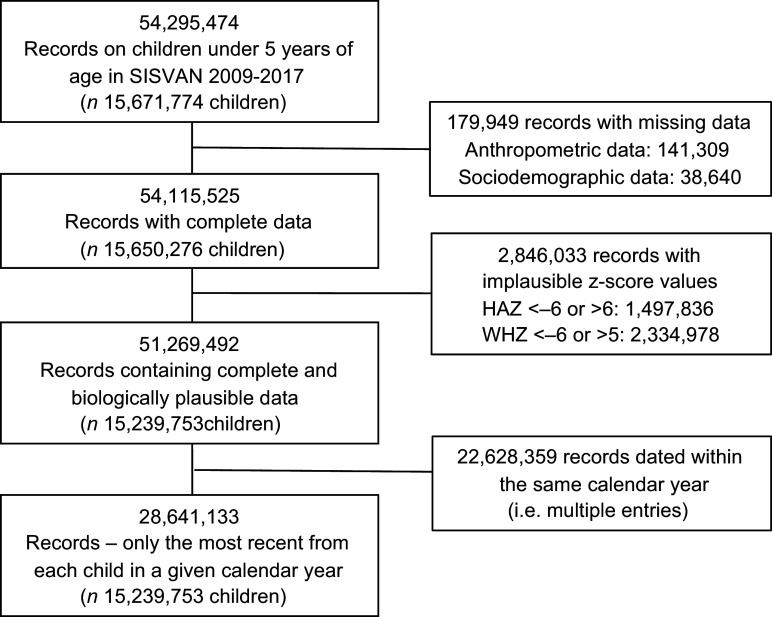
Selection of the study population. SISVAN, 2009–2017

We conducted a time-series study using the utilised nutritional surveillance data on children under 5 years of age who were followed at SUS primary health care services between 2009 and 2017. Individual-level data were obtained from the Food and Nutrition Surveillance System. Data access, processing and analysis were conducted at the Centre for Data and Knowledge Integration for Health (CIDACS), Oswaldo Cruz Foundation (FIOCRUZ)^(18)^. All children for whom at least one recorded entry was identified, containing complete and plausible anthropometric measurements, were included. However, the most recent record per year of follow-up for each child was considered to calculate the annual prevalence of nutritional indicators (Fig. 1).

### Indicators of malnutrition

Data on weight (kg) and height (cm) were extracted from SISVAN records on children under 5 years of age. These measurements are routinely collected by Primary Health Care professionals, who provide healthcare services and conduct nutritional surveillance, as well as monitor compliance with the health conditions established by the Bolsa Família Program (BFP)^(16)^. Technical standards for the collection and analysis of this data in public health services have been established by the Brazilian Ministry of Health^(19)^.

Height-for-age *Z*-scores and weight-for-height *Z*-scores (WHZ) were calculated based on WHO Child Growth Standards curves and then classified in accordance with WHO cut-off points^(20)^. Height-for-age *Z*-score < -6 or >6 and WHZ < -6 or >5 were considered implausible and excluded from the analysis^(21)^. The following indicators of child malnutrition were considered: stunting (height-for-age *Z*-score < -2), wasting (WHZ < -2), overweight/obesity (WHZ > 2) and double burden (height-for-age *Z*-score < -2 and WHZ > 2).

### Demographic and socio-economic variables

The following demographic and socio-economic data were obtained from SISVAN: sex (female/male); age (0–5 months/6–23 months/24–59 months); race/skin colour (white/black/mixed-race/Asian descent/indigenous/not given); traditional community membership – social groups struggling for social basic rights and for territory, access to natural resources and recognition in public policies adequate to their needs, including indigenous groups and Quilombo communities descended from African Brazilian fugitive slave (not declared member/declared member); conditional cash transfer program – Bolsa Familia (not recipient/recipient) and geographic region of residence (North/Northeast/Central-West/Southeast/South). Indicators specific to each individual’s municipality of residence, based on data from the 2010 Demographic Census conducted by the Brazilian Institute of Geography and Statistics, were obtained in accordance with Brazilian Institute of Geography and Statistics municipal codes and integrated into the dataset. All indicators were obtained from the Brazilian Institute of Geography and Statistics Automatic Recovery System (SIDRA)^(22)^ and from the Atlas of Human Development in Brazil^(23)^. The following indicators were analysed: municipality population size (very small ≤20 000 inhabitants/small 20,001–50,000 inhabitants/medium 50,001–100,000 inhabitants/large >100 001 inhabitants), Municipal Human Development Index (MHDI) (very low 0·00–0·49/low 0·50–0·59/medium 0·60–0·69/high 0·70–0·79/very high 0·80–1·00) and Gini index (quintiles).

### Statistical analysis

The prevalence of stunting, wasting, overweight/obesity and double burden of malnutrition were annually estimated by demographic and socio-economic variables. Prais–Winsten estimations were used to analyse temporal trends in prevalence. This generalised linear regression method has been widely employed to correct for serial correlations in time series^(24)^. The annual prevalence for each malnutrition indicator was converted to a logarithmic scale to reduce the heterogeneity of variance in the regression model. Log scale-transformed prevalence values were defined as dependent variables, while the year of follow-up was defined as an independent variable. Annual percent change and respective 95 % CI were calculated according to the formula: Annual percent change = (–1 + 10^*β*^) × 100, where *β* is the coefficient from Prais–Winsten regression^(24)^. Because the increase in case notification and the potential improvements in access and quality of health care for children may explain part of the temporal trend of child malnutrition outcomes, all analyses were adjusted for the variation in SISVAN coverage. As in previous studies^(25)^, SISVAN coverage was calculated based on the number of children under 5 years with a nutritional status record in SISVAN divided by the population under 5 years who are SUS users, multiplied by 100. All data were processed and analysed using Stata software version 15.1 (Stata Corp.).

## Results

The study included a total of 15,239,753 children, 28,641,133 unique records/child and per year of follow-up were used for prevalence calculations for whom at least one SISVAN record between 2009 and 2017 contained complete and plausible data (Fig. 1). The number of children in the SISVAN database varied annually, from 2 050 117 in 2009 to 4 925 581 in 2017 (see online Supplemental Table 1). Children were predominantly aged between 24 and 59 months, recipient of BFP benefits, resided in the Northeast region, in municipalities with ≤ 20 000 inhabitants, with high MHDI and low income inequality (Gini index) values. These characteristics were observed to be consistent over time.

**Fig. 2:**
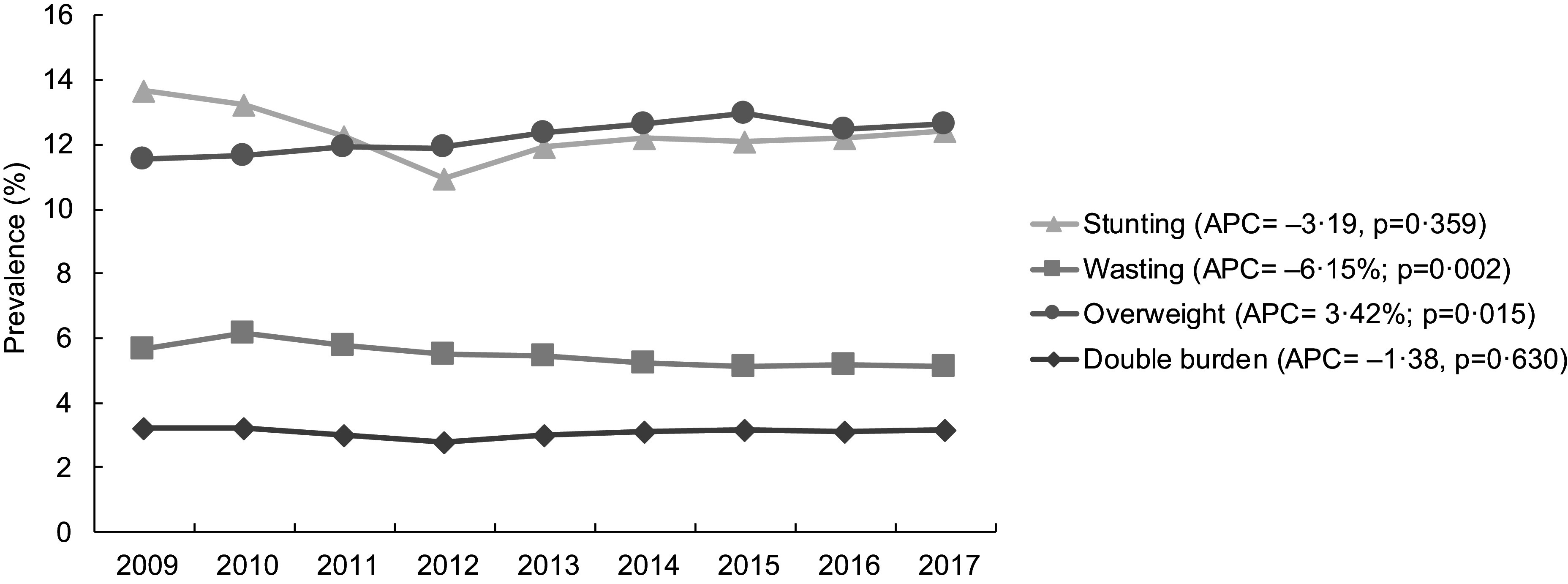
Prevalence of stunting, wasting, overweight/obesity and double burden of malnutrition in children under 5 years old. SISVAN, 2009–2017. APC: annual percentage change. Adjusted for the variation in SISVAN coverage

The overall prevalence of stunting decreased from 13·7 % to 12·4 % between 2009 and 2017; however, this reduction was not statistically significant. The prevalence of wasting decreased significantly from 5·7 % in 2009 to 5·1 % in 2017, an annual variation of –6·2 % (*P* = 0·002). The prevalence of overweight (overweight/obesity) tended to increase over time (3·4 %, *P* = 0·015), rising from 11·6 % in 2009 to 12·6 % in 2017. The prevalence of DBM (simultaneous occurrence of short stature and overweight) ranged around 3 % and remained stable over time (Fig. 2).

The annual prevalence of stunting was predominantly higher among children aged <24 months, who were declared as traditional community’s members, were black/mixed-race/Asian descent/indigenous and resided in the North of Brazil in municipalities with low MHDI and high Gini coefficient values (Table 1). Significant reduction in the prevalence of stunting was observed among those who declared as traditional community’s members (–10·1 %, *P* = 0·003) and were residents in municipalities with very low MHDI (–5·4 %, *P* = 0·036). By contrast, an increasing trend in stunting was observed in municipalities with a very high MHDI value (10·6 %, *P* = 0·035).

**Table 1:** Prevalence of stunting by socio-demographic variables. SISVAN, 2009–2017

	2009	2010	2011	2012	2013	2014	2015	2016	2017	APC (%)	95 % CI		*P*-value	*R* ^2^
Overall	13·7	13·2	12·2	11·0	11·9	12·2	12·1	12·2	12·4	−3·2	−10·6	4·9	0·359	0·97
Sex														
Female	12·8	12·3	11·6	10·7	11·1	11·2	11·0	11·2	11·5	−3·5	−9·5	2·9	0·225	0·99
Male	14·6	14·1	13·0	11·4	13·0	13·5	13·3	13·2	13·4	−2·5	−11·2	7·2	0·539	0·94
Age														
0–5 months	17·6	17·0	17·1	17·0	17·5	17·0	15·1	13·7	15·7	0·1	−1·1	1·4	0·831	0·99
6–23 months	17·8	17·7	15·7	13·4	16·6	16·9	16·7	15·9	16·6	−2·3	−13·2	10·0	0·650	0·80
24–59 months	12·4	11·9	11·2	10·0	10·3	10·4	10·3	10·6	10·7	−4·7	−11·5	2·5	0·157	0·99
Race/skin colour														
White	10·4	9·3	8·8	8·9	10·0	10·3	10·2	10·0	10·0	1·7	−6·6	10·8	0·649	0·95
Black	13·0	11·7	10·7	10·9	12·2	12·0	12·0	12·3	12·3	−0·2	−8·5	8·9	0·966	0·95
Mixed-race	15·1	13·1	11·6	10·5	11·6	14·3	15·2	15·1	13·0	−1·9	−18·7	18·4	0·814	0·91
Asian descent	14·6	13·1	13·3	13·1	14·5	13·6	11·2	10·6	14·3	1·8	−4·9	8·9	0·548	1·00
Indigenous	30·1	29·8	26·1	26·5	29·7	30·8	30·4	29·9	30·1	1·4	−6·6	10·2	0·688	0·97
Not given	18·2	15·8	14·1	11·4	9·6	8·8	8·9	9·5	9·7	−19·1	−31·5	−4·5	0·021	0·96
Traditional communities														
Not declared member	13·7	13·2	12·2	11·0	11·9	12·2	12·1	12·2	12·5	−3·2	−10·7	5·0	0·370	0·97
Declared member	15·1	15·2	14·4	13·1	12·9	12·9	11·4	10·5	9·3	−10·1	−14·7	−5·1	0·003	0·94
Cash transfer recipient														
Not recipient	11·5	10·8	10·2	9·4	9·8	9·8	9·8	9·6	9·7	−5·2	−10·4	0·3	0·060	0·99
Recipient	15·0	14·3	13·3	11·8	12·8	13·1	13·0	13·5	13·6	−3·3	−11·3	5·6	0·391	0·97
Region of residence														
North	22·6	20·9	20·2	17·8	20·1	19·7	17·6	17·7	18·3	−4·6	−11·0	2·3	0·150	0·78
Northeast	15·7	14·6	13·5	11·6	12·2	12·5	12·4	12·6	12·8	−6·3	−15·0	3·2	0·151	0·98
Central-West	11·2	11·6	11·1	10·5	10·1	11·3	11·0	11·3	11·5	−2·9	−7·9	2·5	0·230	0·99
Southeast	9·9	9·8	9·2	8·8	9·9	10·3	10·4	10·8	10·8	2·7	−4·5	10·4	0·402	0·96
South	10·0	9·6	8·7	8·4	8·9	9·1	9·5	9·2	9·0	−3·7	−9·9	2·8	0·206	0·98
Population size*														
Very small (≤20 000 ihn.)	13·3	12·8	11·8	10·6	11·3	11·5	11·7	11·9	11·7	−4·6	−11·5	2·9	0·182	0·98
Small (20 001–50 000 ihn.)	15·4	14·5	13·5	12·0	12·9	13·5	13·1	13·1	13·2	−3·9	−11·5	4·3	0·278	0·97
Medium (50 001–100 000 ihn.)	14·6	14·4	13·4	12·0	13·2	13·7	13·3	13·0	13·6	−2·1	−9·4	5·8	0·533	0·94
Large (>100 001 ihn.)	12·2	11·9	11·0	9·9	11·1	11·3	11·2	11·6	11·9	−1·4	−9·3	7·2	0·694	0·95
Municipal HDI*														
Very low (0·000–0·499)	28·4	29·5	27·3	24·7	26·5	26·0	24·1	24·0	24·5	−5·4	−10·1	−0·5	0·036	0·97
Low (0·500–0·599)	18·4	17·5	16·3	14·2	14·9	15·4	15·0	15·2	15·2	−5·7	−13·2	2·4	0·133	0·98
Medium (0·600–0·699)	14·0	13·1	12·3	10·8	11·7	11·8	11·9	12·1	12·4	−4·6	−12·6	4·2	0·239	0·96
High (0·700–0·799)	10·5	10·3	9·5	9·1	9·8	10·3	10·2	10·3	10·3	0·1	−6·4	7·1	0·968	0·98
Very high (0·800–1·000)	8·5	8·2	7·9	8·2	10·1	10·0	11·2	11·9	12·2	10·6	1·0	21·1	0·035	0·88
Gini index*														
Q1 lowest	10·3	10·1	9·4	8·9	9·4	9·7	10·0	10·2	10·2	−0·9	−7·2	5·7	0·739	0·98
Q2	12·3	11·6	10·6	9·6	10·3	10·6	10·5	10·3	10·5	−4·7	−12·0	3·3	0·196	0·98
Q3	13·6	13·1	12·1	11·0	11·8	12·0	11·7	11·9	12·2	−3·1	−10·5	4·9	0·372	0·98
Q4	16·6	15·5	14·6	12·4	13·6	13·9	13·3	13·4	13·8	−5·8	−14·2	3·4	0·168	0·92
Q5 highest	18·2	17·5	16·1	14·2	15·5	15·8	15·3	15·7	15·8	−4·3	−12·1	4·3	0·256	0·97

APC: annual percentage change; *R* ^2^: coefficient of determination; HDI: human development index.

* Socio-demographic characteristics of the municipality of residence. 2010 Demographic Census, Brazilian Institute of Geography and Statistics.

Adjusted for the variation in SISVAN coverage.

The prevalence of wasting was higher mainly among black/mixed-race/Asian descent/indigenous children aged 24–59 months who were recipients of BFP, resided in Northeast and North regions and in municipalities with lower MHDI and Gini index scores (Table 2). A trend of decreasing prevalence was also observed for this indicator in children of both sexes, female (–5·8 %, *P* = 0·003) and male (–6·3 %, *P* = 0·002), aged between 24 and 59 months (–5·7 %, *P* = 0·005); in those who declared (–14·2 %, *P* = 0·001) or not (–5·9 %, *P* = 0·002) as traditional community’s members; were black (–5·7 %, *P* = 0·013) and were recipients (–6·7 %, *P* = 0·007) or not of BFP benefits (–5·1 %, *P* = 0·001). The prevalence of wasting reduced across almost all regions of the country, especially in the Northeast (–8·0 %, *P* = 0·004) and the Central-West (–6·8 %, *P* = 0·002); in municipalities with variable population sizes, mostly small (–7·0 %, *P* = 0·001) and small-sized (–7·2 %, *P* = 0·001); with low and medium MHDI scores, especially low MHDI (–8·4 %, *P* = 0·005) and across all quintiles of the Gini index.

**Table 2:** Prevalence of wasting by socio-demographic variables. SISVAN, 2009–2017

	2009	2010	2011	2012	2013	2014	2015	2016	2017	APC (%)	95 % CI		*P*-value	*R* ^2^
Overall	5·7	6·2	5·8	5·5	5·5	5·2	5·1	5·2	5·1	−6·2	−9·0	−3·2	0·002	0·99
Sex														
Female	5·6	6·0	5·7	5·4	5·4	5·2	5·1	5·1	5·0	−5·8	−8·5	−2·9	0·003	0·99
Male	5·8	6·3	5·9	5·6	5·5	5·3	5·2	5·2	5·2	−6·3	−9·3	−3·3	0·002	0·99
Age														
0–5 months	5·2	4·9	4·9	5·0	4·8	4·9	5·1	5·1	4·9	−1·8	−4·0	0·4	0·096	1·00
6–23 months	4·0	4·3	3·7	3·4	3·9	3·8	3·8	3·8	3·8	−3·0	−10·8	5·4	0·400	0·09
24–59 months	6·2	6·7	6·3	6·1	6·0	5·7	5·6	5·7	5·6	−5·7	−8·9	−2·5	0·005	0·98
Race/skin colour														
White	4·2	4·3	3·8	3·8	4·0	4·1	4·1	4·1	4·1	−0·7	−6·7	5·6	0·792	0·85
Black	6·0	6·4	5·8	5·5	5·8	5·4	5·4	5·6	5·6	−5·7	−9·6	−1·7	0·013	0·98
Mixed-race	6·6	7·1	6·6	6·1	5·5	5·2	5·2	5·7	5·9	−6·9	−16·0	3·1	0·138	0·95
Indigenous	5·5	5·1	5·2	4·6	5·3	5·4	4·9	4·7	4·5	−0·5	−7·4	7·0	0·878	0·73
Asian descent	6·1	6·3	5·3	5·5	6·1	6·0	6·2	6·1	5·6	−2·1	−10·1	6·7	0·571	0·72
Not given	6·2	6·5	6·3	6·5	6·1	5·4	4·2	3·9	3·8	−10·5	−19·6	−0·3	0·046	0·95
Traditional communities														
Not declared member	5·7	6·2	5·8	5·5	5·5	5·2	5·2	5·2	5·1	−5·9	−8·7	−3·1	0·002	0·99
Declared member	5·6	5·9	5·6	5·2	4·5	4·3	4·1	3·9	3·6	−14·2	−19·4	−8·7	0·001	0·94
Cash transfer recipient														
Not recipient	4·4	4·6	4·3	4·2	4·2	4·1	3·9	3·9	3·7	−5·1	−7·1	−3·0	0·001	1·00
Recipient	6·5	6·8	6·5	6·3	6·0	5·6	5·6	5·8	5·7	−6·7	−10·6	−2·7	0·007	0·91
Region of residence														
North	6·8	6·9	6·7	6·4	6·6	6·0	5·9	5·9	6·2	−5·0	−8·6	−1·2	0·019	0·97
Northeast	7·1	7·7	7·3	6·9	6·7	6·2	6·1	6·3	6·0	−8·0	−12·0	−3·9	0·004	0·93
Central-west	6·0	6·2	6·0	5·7	5·4	5·2	5·0	5·1	5·0	−6·8	−9·9	−3·6	0·002	0·98
Southeast	4·4	4·7	4·4	4·3	4·4	4·5	4·4	4·4	4·3	−1·9	−4·7	1·1	0·174	0·99
South	3·2	3·4	3·1	3·1	3·0	3·0	2·9	3·0	2·9	−4·1	−6·0	−2·1	0·003	1·00
Population size*														
Very small (≤20 000 ihn.)	6·0	6·4	6·1	5·8	5·6	5·3	5·4	5·5	5·3	−7·0	−10·0	−4·0	0·001	0·97
Small (20 001–50 000 ihn.)	6·4	7·0	6·5	6·2	6·1	5·8	5·8	5·9	5·6	−7·2	−9·9	−4·5	0·001	0·99
Medium (50 001–100 000 ihn.)	6·0	6·5	6·1	5·8	5·7	5·7	5·5	5·4	5·3	−5·5	−8·0	−2·9	0·002	1·00
Large (>100 001 ihn.)	4·4	4·9	4·5	4·5	4·5	4·3	4·2	4·3	4·4	−2·8	−6·4	0·9	0·112	0·98
Municipal HDI*														
Very low (0·000–0·499)	8·0	7·8	6·8	6·6	7·6	7·5	7·5	6·4	6·8	−3·6	−12·7	6·4	0·400	0·68
Low (0·500–0·599)	7·6	8·2	7·8	7·5	7·1	6·5	6·5	6·8	6·4	−8·4	−12·9	−3·7	0·005	0·83
Medium (0·600–0·699)	6·3	6·7	6·3	6·1	5·9	5·6	5·6	5·7	5·7	−6·4	−9·8	−3·0	0·004	0·84
High (0·700–0·799)	4·0	4·4	4·0	3·9	4·0	4·0	3·9	4·0	3·9	−2·6	−5·4	0·2	0·065	0·99
Very high (0·800–1·000)	3·1	3·5	3·3	3·7	4·0	3·9	3·7	3·5	3·6	11·6	7·1	16·2	0·001	0·99
Gini index*														
Q1 lowest	4·5	4·9	4·6	4·4	4·3	4·3	4·3	4·3	4·3	−4·9	−7·7	−2·1	0·006	0·99
Q2	5·6	5·8	5·3	5·1	5·1	4·9	4·7	4·8	4·7	−7·6	−10·1	−5·1	0·000	0·99
Q3	5·9	6·2	6·0	5·7	5·5	5·3	5·3	5·4	5·3	−6·1	−9·2	−2·8	0·004	0·86
Q4	6·7	7·2	6·8	6·4	6·4	6·0	5·9	5·9	5·7	−7·1	−10·0	−4·2	0·001	0·99
Q5 highest	6·4	7·1	6·6	6·4	6·3	5·9	5·6	5·6	5·8	−5·8	−10·4	−1·0	0·025	0·95

APC: annual percentage change; *R* ^2^: coefficient of determination; HDI: human development index.

* Socio-demographic characteristics of the municipality of residence. 2010 Demographic Census, Brazilian Institute of Geography and Statistics.

Adjusted for the variation in SISVAN coverage.

A higher prevalence of overweight was observed in male children aged 6–23 months, who were recipients of BFP and resided in the Northeast and South regions (Table 3). Significant increase in the prevalence of this indicator was seen in females (3·8 %, *P* = 0·015), children aged 24–59 months (2·8 %, *P* = 0·035), who did not declare (3·4 %, *P* = 0·016) and declared as traditional community’s members (3·1 %, *P* = 0·032), were white (6·0 %, *P* = 0·008), indigenous (5·5 %, *P* = 0·024) and black (10·4 %, *P* = 0·002), and were recipients of BFP (3·5 %, *P* = 0·015). An increasing trend in the prevalence of overweight was also observed across almost all regions, especially in the North (6·1 %, *P* = 0·015) and Southeast (5·6 %, *P* = 0·007); in municipalities of almost all population sizes, especially small (5·0 %, *P* = 0·002); almost all MHDI levels, especially very high (8·7 %, *P* = 0·003) and across all quintiles of the Gini index, mainly in municipalities with higher inequality (Q5: 6·3 %, *P* = 0·003).

**Table 3:** Prevalence of overweight (overweight/obesity) by socio-demographic variables. SISVAN, 2009–2017

	2009	2010	2011	2012	2013	2014	2015	2016	2017	APC (%)	95 % CI		*P*-value	*R* ^2^
Overall	11·6	11·7	12·0	11·9	12·4	12·7	13·0	12·5	12·6	3·4	0·9	6·0	0·015	0·90
Sex														
Female	10·9	11·0	11·6	11·8	11·8	12·0	12·2	11·8	12·0	3·8	1·0	6·7	0·015	0·87
Male	12·3	12·3	12·4	12·1	13·1	13·5	13·8	13·2	13·3	3·2	−1·1	7·6	0·120	0·96
Age														
00–05 months	11·0	11·3	11·2	10·9	11·4	10·7	11·8	10·5	11·5	−0·5	−2·0	1·1	0·487	1·00
06–23 months	15·6	16·1	16·3	15·7	16·3	16·5	16·4	15·1	15·7	1·7	−1·1	4·5	0·192	1·00
24–59 months	10·5	10·5	10·9	11·0	11·2	11·5	11·9	11·7	11·5	2·8	0·3	5·5	0·035	1·00
Race/skin colour														
White	11·9	11·5	11·9	12·2	13·0	12·9	13·3	12·4	12·6	6·0	2·2	9·9	0·008	1·00
Black	10·7	10·2	11·2	12·2	12·4	12·6	13·0	12·4	12·5	10·4	5·2	16·0	0·002	0·99
Mixed-race	11·7	10·9	10·9	11·0	11·5	13·7	14·5	13·5	12·1	4·1	−7·7	17·5	0·446	0·94
Asian descent	11·0	10·8	14·2	13·7	13·1	12·7	11·6	11·5	13·7	7·9	−5·0	22·5	0·195	0·46
Indigenous	10·8	9·8	10·4	10·9	11·8	10·9	10·0	9·9	10·1	5·5	1·0	10·2	0·024	0·99
Not given	10·4	12·7	12·3	11·4	10·9	10·8	11·7	11·5	11·7	−3·7	−10·6	3·8	0·266	0·92
Traditional communities														
Not declared member	11·6	11·7	12·0	11·9	12·4	12·7	13·0	12·5	12·7	3·4	0·9	6·0	0·016	0·96
Declared member	11·0	11·3	11·2	11·9	11·8	11·7	10·9	10·5	10·8	3·1	0·4	5·9	0·032	1·00
Cash transfer recipient														
Not recipient	11·1	11·1	11·4	11·3	11·4	11·4	12·0	11·5	11·7	1·6	0·0	3·2	0·054	1·00
Recipient	11·8	11·9	12·2	12·3	12·8	13·1	13·3	13·0	13·0	3·5	0·9	6·1	0·015	1·00
Region of residence														
North	9·4	9·0	9·1	9·4	10·1	10·6	10·4	10·7	10·4	6·1	1·6	10·8	0·015	0·98
Northeast	12·7	12·8	13·0	13·0	13·2	14·1	14·4	14·2	14·4	4·6	1·8	7·4	0·006	0·99
Central-West	10·5	10·9	10·7	10·9	10·9	11·2	11·5	11·0	10·8	1·3	−1·7	4·4	0·333	0·05
Southeast	10·8	11·0	11·7	11·6	12·3	12·0	12·4	11·5	11·7	5·6	2·1	9·1	0·007	1·00
South	11·6	11·9	11·9	11·7	12·6	12·2	13·0	12·0	12·4	3·0	0·1	6·0	0·044	1·00
Population size*														
Very small (≤20 000 ihn.)	11·9	11·9	12·2	11·9	12·4	12·7	13·1	12·8	12·9	2·6	0·3	5·1	0·035	0·85
Small (20 001–50 000 ihn.)	11·5	11·7	12·1	12·1	12·5	13·1	13·1	12·7	13·0	5·0	2·7	7·4	0·002	0·89
Medium (50 001–100 000 ihn.)	12·1	11·7	12·1	12·4	12·7	13·0	13·1	12·4	12·6	4·6	1·8	7·4	0·006	1·00
Large (>100 001 ihn.)	11·0	11·3	11·6	11·6	12·0	12·1	12·7	12·1	12·1	2·1	−1·4	5·7	0·199	0·99
Municipal HDI*														
Very low (0·000–0·499)	13·2	12·2	12·4	9·5	11·2	11·2	10·4	11·3	10·2	−7·8	−15·6	0·8	0·068	0·96
Low (0·500–0·599)	11·7	11·7	11·6	11·4	11·8	12·7	12·8	12·7	12·9	3·9	0·0	8·0	0·049	0·99
Medium (0·600–0·699)	12·0	12·0	12·4	12·5	12·9	13·2	13·5	13·1	13·3	4·3	2·2	6·4	0·002	0·98
High (0·700–0·799)	11·2	11·5	11·8	11·8	12·4	12·2	12·7	12·1	12·1	4·3	1·4	7·3	0·010	1·00
Very high (0·800–1·000)	10·3	10·1	10·7	11·0	12·0	11·5	12·4	11·1	11·5	8·7	4·2	13·3	0·003	1·00
Gini index*														
Q1 lowest	11·9	12·1	12·4	12·4	12·9	12·9	13·4	12·6	12·8	3·9	1·2	6·6	0·011	1·00
Q2	11·8	12·0	12·2	12·1	12·7	12·8	13·2	12·4	12·7	3·7	0·9	6·6	0·019	1·00
Q3	11·5	11·9	12·1	12·1	12·5	12·9	13·2	12·9	13·1	4·5	2·5	6·5	0·001	0·88
Q4	11·4	11·6	11·7	11·5	11·7	12·4	12·6	12·4	12·6	3·3	0·8	5·9	0·019	0·98
Q5 highest	10·9	10·5	11·2	11·3	11·8	12·2	12·5	12·2	12·1	6·3	3·0	9·7	0·003	0·99

APC: annual percentage change; *R* ^2^: coefficient of determination; HDI: human development index.

* Socio-demographic characteristics of the municipality of residence. 2010 Demographic Census, Brazilian Institute of Geography and Statistics.

Adjusted for the variation in SISVAN coverage.

The prevalence of double burden of malnutrition was seen more frequently in males, age <24 months, indigenous, recipients of BFP, residents of the North or Northeast regions and municipalities with very low MHDI and Gini index scores (Table 4). A reduction in the prevalence of this phenotype was detected among children aged <6 months (–2·8 %, *P* = 0·045), who declared as traditional community’s members (–10·1 %, *P* = 0·049). On the other hand, the prevalence of this indicator increased among children who were indigenous (6·6 %, *P* = 0·028), resided in the Southeast region (7·1 %, *P* = 0·033) and in municipalities with very high MHDI (24·1 %, *P* = 0·001).

**Table 4:** Prevalence of double burden of malnutrition by socio-demographic variables. SISVAN, 2009–2017

	2009	2010	2011	2012	2013	2014	2015	2016	2017	APC (%)	95 % CI		*P*-value	*R* ^2^
Overall	3·2	3·2	3·0	2·8	3·0	3·1	3·2	3·1	3·1	−1·4	−7·8	5·5	0·630	0·86
Sex														
Female	3·0	3·0	2·9	2·8	2·8	2·9	2·9	2·8	2·9	−1·4	−4·6	1·9	0·334	0·96
Male	3·5	3·4	3·1	2·7	3·2	3·4	3·5	3·3	3·3	−1·0	−11·2	10·4	0·830	0·67
Age														
00–05 months	5·0	5·2	4·9	4·9	4·9	4·7	5·0	3·9	4·7	−2·8	−5·5	−0·1	0·045	1·00
06–23 months	5·1	5·3	4·8	4·1	4·9	5·0	5·0	4·3	4·7	−2·8	−12·9	8·5	0·549	0·12
24–59 months	2·6	2·6	2·5	2·3	2·3	2·4	2·4	2·5	2·5	−2·1	−7·4	3·4	0·377	0·94
Race/skin color														
White	2·6	2·3	2·2	2·3	2·7	2·8	2·8	2·6	2·6	4·3	−6·1	15·8	0·367	0·65
Black	2·9	2·5	2·6	2·9	3·1	3·1	3·2	3·1	3·2	8·0	−0·2	16·9	0·055	0·65
Mixed-race	3·6	3·1	2·7	2·5	2·8	3·9	4·3	3·9	3·1	0·2	−22·9	30·3	0·984	0·56
Asian descent	3·3	3·0	4·0	3·8	3·8	3·5	2·6	2·6	3·9	8·0	−11·1	31·1	0·371	0·32
Indigenous	4·5	4·6	4·2	4·4	5·1	5·0	4·3	4·1	4·4	6·6	1·0	12·5	0·028	0·98
Not given	3·7	4·1	3·5	2·6	2·1	2·0	2·2	2·3	2·3	−20·9	−33·4	−6·0	0·016	0·85
Traditional communities														
Not declared member	3·2	3·2	3·0	2·8	3·0	3·1	3·2	3·1	3·2	−1·4	−7·9	5·7	0·643	0·85
Declared member	3·2	3·2	3·2	3·3	3·2	2·9	2·5	2·3	2·0	−10·1	−19·2	0·0	0·049	0·92
Cash transfer recipient														
Not recipient	2·6	2·5	2·4	2·3	2·3	2·3	2·6	2·4	2·4	−5·2	−10·3	0·2	0·056	0·84
Recipient	3·6	3·5	3·3	3·0	3·3	3·4	3·4	3·4	3·5	−1·0	−7·6	6·0	0·726	0·91
Region of residence														
North	4·0	3·8	3·5	3·5	3·9	4·1	3·6	3·5	3·5	0·8	−6·1	8·2	0·797	0·90
Northeast	4·1	4·0	3·7	3·2	3·4	3·6	3·6	3·7	3·7	−2·9	−11·6	6·5	0·463	0·92
Central-west	2·8	3·1	2·8	2·6	2·6	2·9	2·9	2·8	2·8	−3·0	−9·7	4·2	0·341	0·17
Southeast	2·2	2·2	2·3	2·2	2·5	2·6	2·7	2·5	2·5	7·1	0·8	13·7	0·033	0·81
South	2·3	2·3	2·1	2·0	2·3	2·2	2·6	2·4	2·3	−3·6	−10·3	3·6	0·256	0·81
Population size*														
Very small (≤20 000 ihn.)	3·4	3·4	3·2	2·8	3·1	3·1	3·3	3·3	3·2	−3·4	−9·9	3·5	0·261	0·86
Small (20 001–50 000 ihn.)	2·5	3·7	3·6	3·4	3·1	3·3	3·6	3·4	3·4	1·5	−11·4	16·3	0·795	0·67
Medium (50 001–100 000 ihn.)	3·5	3·4	3·1	3·1	3·3	3·5	3·4	3·2	3·4	0·0	−6·9	7·4	0·996	0·86
Large (>100 001 ihn.)	2·5	2·6	2·4	2·2	2·5	2·6	2·7	2·6	2·7	0·7	−6·1	8·1	0·812	0·54
Municipal HDI*														
Very low (0·000–0·499)	5·9	6·5	5·4	3·9	5·1	5·3	4·2	4·4	4·1	−9·7	−22·8	5·6	0·161	0·56
Low (0·500–0·599)	4·4	4·3	4·0	3·5	3·6	3·9	3·8	3·9	3·9	−3·2	−11·3	5·8	0·410	0·93
Medium (0·600–0·699)	3·5	3·4	3·3	3·0	3·2	3·3	3·4	3·3	3·4	−1·6	−8·0	5·2	0·574	0·92
High (0·700–0·799)	2·3	2·4	2·2	2·2	2·4	2·5	2·6	2·5	2·4	1·7	−5·0	8·7	0·575	0·65
Very high (0·800–1·000)	1·6	1·5	1·6	1·9	2·3	2·2	2·7	2·4	2·7	24·1	13·1	36·1	0·001	0·95
Gini index*														
Q1 lowest	2·6	2·6	2·5	2·4	2·6	2·7	2·8	2·7	2·8	1·0	−3·6	5·8	0·636	0·91
Q2	3·1	3·1	2·8	2·6	2·8	2·9	3·0	2·7	2·8	−3·9	−11·0	3·7	0·250	0·82
Q3	3·3	3·3	3·1	2·8	3·1	3·2	3·2	3·3	3·3	−0·4	−7·4	7·2	0·902	0·78
Q4	3·8	3·7	3·5	3·0	3·3	3·5	3·4	3·5	3·6	−2·1	−10·8	7·6	0·606	0·84
Q5 highest	3·8	3·6	3·4	3·2	3·5	3·5	3·5	3·3	3·4	−2·5	−8·7	4·2	0·386	0·88

APC: annual percentage change; *R* ^2^: coefficient of determination; HDI: human development index.

* Socio-demographic characteristics of the municipality of residence. 2010 Demographic Census, Brazilian Institute of Geography and Statistics.

Adjusted for the annual nationwide coverage of SISVAN.

## Discussion

Overall, our findings demonstrate an increasing prevalence of overweight, declining prevalence of wasting and stability in the prevalence of stunting and DBM (concurrent short stature and overweight) among children followed in primary care services in the Brazilian Unified Health System (SUS) between 2009 and 2017. Our trend analysis in accordance with demographic and socio-economic variables indicated a reduction in the prevalence of wasting across most of the studied subgroups, in particular children declared as traditional community’s members (Annual percent change –14·2 %). A reduction in the prevalence of stunting was only observed in children of traditional communities (–10·1 %) and who resided in municipalities with lower MHDI scores. Despite this reduction, the overall prevalence of undernutrition forms (stunting and wasting) remained very high over the almost 10-year study period. Surprisingly, the prevalence of stunting (10·6 %), wasting (11·6 %) and DBM (24·1 %) increased in children who lived in municipalities with higher MHDI.

Stunting and wasting are distinct undernutrition phenomena. Stunting or linear growth deficit is generally considered to reflect long-term exposure to nutritional stresses. Unlike linear growth deficit, wasting or low weight for height appears to be short term in nature. Weight-for-height shifts are often less extreme, perhaps because weight for height is more tightly biologically controlled^(26)^. However, linear growth and weight faltering are both rooted in poverty and are likely to result from a mixture of environmental determinants, including infectious exposures, dietary and socio-economic factors^(27,26)^. Therefore, it is possible that contextual interventions that promote linear growth will also prevent wasting. The obvious failure to overcome the plight of child malnutrition, an aspiration strived for in the first decade of this millennium, is a reflection of arrested economic growth and income redistribution policies, as well as declining investment in the universal access to education, health and proper sanitation services for all Brazilian families^(28,29)^. This phenomenon is notable in other Latin American countries where health inequalities remain a major concern^(30)^. While the prevalence of stunting increased in Eastern Africa, North Africa and the Caribbean experienced only modest improvements. Very little progress has been made in Western Africa and Central America^(31)^. Child malnutrition remains a major public health concern among impoverished populations, with potentially disastrous consequences for child growth, development and even survival^(7,3)^.

Our results indicate that Brazil faces a nutritional paradox, as the prevalence of overweight increased during the period studied. Of note, differences in the prevalence of overweight between the richest and poorest areas were small, especially in the final years studied. Our findings constitute a perverse example where a reduction in inequalities was due to the worsening of nutritional status and disproportionate increase in overweight among the most vulnerable groups (black children, who were recipients of BFP benefits, resided in the poorer North/Northeast regions in less-developed municipalities with high inequality). While the prevalence of overweight and obesity among children and adolescents is growing in low- and middle-income countries, some country-specific analysis have suggested recent plateaus, or even decreases, in the prevalence of excess weight, especially in high-income countries^(32)^. Increasing trends in overweight and obesity in low- and middle-income countries have been attributed to ongoing demographic and nutritional transitions, mainly arising from urbanisation and globalisation leading to the adoption of unhealthy lifestyles, reductions in physical activity and highly caloric and low-nutrition diets^(33,6)^. In the Brazilian population, inadequate diet is a primary risk factor related to overweight/obesity, and could have contributed to increasing tendency towards overweight observed in the present study^(34)^.

This information serves as an alert regarding inadequacies in the nutritional quality of Brazilians’ diets, especially poorer populations, who employ alternatives to suppress hunger, including adopting inappropriate practices such as overdiluting milk/juice, reducing portion sizes to stretch limited supplies, consequently lowering nutritional intake and restricting the purchase of healthier food options, which leads to increased consumption of cheaper and less nutritious alternatives. These practices can lead to a higher risk of DBM, in addition to other adverse health outcomes^(35,36)^. While the prevalence of DBM was found to be stable over the studied period, it reached just over 5 % in children aged 2 years or younger. Although this phenomenon has been well-documented in households and communities throughout the stages of life^(37)^, very little is known with respect to individuals, especially young children. The underlying basis of DBM is not yet fully understood, but some studies have suggested it is particularly related to inadequate nutrition during the period following conception, resulting in restricted intrauterine growth—which might entail delayed linear growth—and subsequent exposure to ‘obesogenic environments’^(38,39)^.

Unfortunately, efforts aimed at reducing child malnutrition and improving the quality of life of future generations have not continued to be policy priorities in Brazil. Several public policies implemented in the early 2000s enabled the country to address historical undernutrition, resulting in the removal of Brazil from the United Nations World Food Program’s Hunger Map in 2014^(40)^. One such initiative was the BFP, a conditional cash transfer program that contributed to meeting poverty reduction goals in all Brazilian states^(41)^. Conditional cash transfers, made directly to the program’s beneficiaries, create conditions that promote the reduction in income inequality and advancement in the HDI. It has been estimated that the cash transfer mechanism employed by the BFP was responsible for lifting over 20 million people out of poverty throughout Brazil^(42)^.

A reverse in this trend of reducing child malnutrition has been followed by increases in poverty and inequality^(43)^, making Brazil more difficult to achieve the United Nations’ Sustainable Development Goals by 2030^(12)^. Since 2014, the country has endured a significant economic downturn, accompanied by heightened political instability. Social policies positively impacting income distribution, poverty reduction and the food and nutrition security of Brazilians withered after 2016^(44)^. The slowdown in economic growth, together with governmental economic austerity policies and the passage of a constitutional amendment that placed a 20-year limit on increases in Brazil’s public spending in 2016, has further undone social policies that resulted in a reduction in social inequality and income redistribution between 2003 and 2014^(45)^. This scenario has not only affected the Unified Health System (SUS) and the Unified Social Assistance System (SUAS), but also the National Food and Nutrition Security System (SISAN), thereby exacerbating the poverty and social vulnerability that afflicts many Brazilian families^(44,45)^.

In order to more rapidly abate all forms of childhood malnutrition in Brazil, it will be necessary to secure political commitments seeking the continuance of national policies, strategies and interventions in a regular and sustained manner in coming years^(46)^. Among these initiatives, we highlight the breastfeeding and complementary feeding strategy (Amamenta e Alimenta Brasil), the strategy for child food fortification with micronutrients (NutriSUS), the school health program (Programa Saúde na Escola), the dietary guidelines for Brazilian infant and preschool children, the Bolsa Família’s health conditions (monitoring of nutritional status, vaccination and prenatal visits) and the qualification of maternal, prenatal and child health care. In addition, it may be useful to learn from successful actions implemented by other countries that have adopted an integrated strategy to address all forms of malnutrition by focusing on interventions during pregnancy and in the first 2 years of life, promoting linear growth as well as the regulation of food environments that will contribute to the prevention of excessive weight gain on a population level^(47)^.

### Strengths and limitations

A major strength of this study is its use of an unprecedented volume of data on the population served by public primary care services in Brazil. Moreover, our results demonstrate how four outcomes of child malnutrition in the country have evolved from 2009 to 2017, which has been unclear since studies reporting on data through 2006 revealed significant reductions in stunting in the early 2000s. Importantly, the present results indicate an interruption in that progress. However, some limitations must be considered. As with all types of secondary data, whose collection was not primarily designed for research purposes, limitations with regard to incompleteness, underestimation and classification bias must be recognised. In an attempt to improve accuracy, only records with complete weight and height information containing biologically plausible values were included in the study. Of all the records considered, just 0·3 % were found to be incomplete and 5·3 % were deemed implausible. A large annual variation was detected in the distribution of the study population according to the ‘skin colour/race’ variable (Table 1). This variation was mainly observed in mixed-race and Asian descent categories, indicating the possibility of classification error or improper form completion. It is worth noting that most of the population studied herein that is registered in SISVAN are recipients of BFP benefits (∼ 68 %); nutritional surveillance in children aged 0–7 years is incorporated into the program’s directives. That indicates an overrepresentation of the poorest population of smaller or rural municipalities and an underrepresentation of the urban middle- and upper-class population. Accordingly, any interpretation or generalisation of the results must be made with due caution.

## Conclusion

The present results detail a nutrition transition affecting children in Brazil. Recent patterns of stable prevalence of stunting and increasing prevalence of overweight in Brazilian children occur similarly to what has been observed in other low- and middle-income countries. The precarious nutritional conditions seen in more vulnerable populations represent a call to reinforce and expand Brazil’s social protection policies. The regular practice of food and nutrition surveillance is possible as demonstrated herein and essential to protecting children suffering from diverse forms of malnutrition.

## Supplementary material

For supplementary material accompanying this paper visit https://doi.org/10.1017/S1368980021004882. click here to view supplementary material
