# Supplementary material for: Time trends and social inequalities in child malnutrition: nationwide estimates from Brazil’s food and nutrition surveillance system, 2009–2017
Source: Public Health Nutr. 2021 Dec 17;25(12):3366–76. doi: 10.1017/S1368980021004882 (PMC9991727; doi:10.1017/S1368980021004882)
Supplement: Supplementary file 1 [file S1368980021004882sup.zip › S1368980021004882sup001.docx]

**Supplementary Table 1**. Sociodemographic characteristics of the study population. SISVAN, 2009-2017.

|  | **2009** | | **2010** | | **2011** | | **2012** | | **2013** | | **2014** | | **2015** | | **2016** | | **2017** | |
| --- | --- | --- | --- | --- | --- | --- | --- | --- | --- | --- | --- | --- | --- | --- | --- | --- | --- | --- |
|  | **n** | **%** | **n** | **%** | **n** | **%** | **n** | **%** | **n** | **%** | **n** | **%** | **n** | **%** | **n** | **%** | **n** | **%** |
| Overall | 2,050,117 |  | 2,661,321 |  | 2,537,800 |  | 2,380,550 |  | 2,613,038 |  | 1,795,949 |  | 4,728,220 |  | 4,925,581 |  | 4,948,557 |  |
| **Sex** |  |  |  |  |  |  |  |  |  |  |  |  |  |  |  |  |  |  |
| Female | 1,008,591 | 49.2 | 1,304,626 | 49.0 | 1,307,396 | 51.5 | 1,366,173 | 57.4 | 1,438,968 | 55.1 | 964,264 | 53.7 | 2,445,858 | 51.7 | 2,444,626 | 49.6 | 2,429,630 | 49.1 |
| Male | 1,041,526 | 50.8 | 1,356,695 | 51.0 | 1,230,404 | 48.5 | 1,014,377 | 42.6 | 1,174,070 | 44.9 | 831,685 | 46.3 | 2,282,362 | 48.3 | 2,480,955 | 50.4 | 2,518,927 | 50.9 |
| **Age** |  |  |  |  |  |  |  |  |  |  |  |  |  |  |  |  |  |  |
| 00-05 months | 79,225 | 3.9 | 71,228 | 2.7 | 90,843 | 3.6 | 104,220 | 4.4 | 93,372 | 3.6 | 65,324 | 3.6 | 235,512 | 5.0 | 304,214 | 6.2 | 174,812 | 3.5 |
| 06-23 months | 420,665 | 20.5 | 542,529 | 20.4 | 483,267 | 19.0 | 452,912 | 19.0 | 585,444 | 22.4 | 433,263 | 24.1 | 1,150,474 | 24.3 | 1,319,872 | 26.8 | 1,324,438 | 26.8 |
| 24-59 months | 1,550,227 | 75.6 | 2,047,564 | 76.9 | 1,963,690 | 77.4 | 1,823,418 | 76.6 | 1,934,222 | 74.0 | 1,297,362 | 72.2 | 3,342,234 | 70.7 | 3,301,495 | 67.0 | 3,449,307 | 69.7 |
| **Race/skin colour** |  |  |  |  |  |  |  |  |  |  |  |  |  |  |  |  |  |  |
| White | 706,000 | 34.4 | 637,113 | 23.9 | 573160 | 22.6 | 592,044 | 24.9 | 707,883 | 27.1 | 535,139 | 29.8 | 1,457,150 | 30.8 | 1,540,260 | 31.3 | 1,490,752 | 30.1 |
| Black | 79,097 | 3.9 | 68,468 | 2.6 | 53,648 | 2.1 | 58,171 | 2.4 | 74,651 | 2.9 | 56,383 | 3.1 | 149,747 | 3.2 | 147,599 | 3.0 | 150,311 | 3.0 |
| Mixed-race | 1,022,277 | 49.9 | 889,907 | 33.4 | 612,078 | 24.1 | 412,899 | 17.3 | 272,719 | 10.4 | 239,275 | 13.3 | 1,324,042 | 28.0 | 1,781,693 | 36.2 | 1,613,709 | 32.6 |
| Asian descent | 71,123 | 3.5 | 87,472 | 3.3 | 158,036 | 6.2 | 415,435 | 17.5 | 952,352 | 36.5 | 728,675 | 40.6 | 1,447,726 | 30.6 | 1,076,724 | 21.9 | 1,363,006 | 27.5 |
| Indigenous | 14,957 | 0.7 | 14,185 | 0.5 | 10476 | 0.4 | 11,395 | 0.5 | 18,600 | 0.7 | 16,760 | 0.9 | 50,109 | 1.1 | 50,680 | 1.0 | 53,731 | 1.1 |
| Not given | 156,663 | 7.6 | 964,176 | 36.2 | 1,130,402 | 44.5 | 890,606 | 37.4 | 586,833 | 22.5 | 219,717 | 12.2 | 299,446 | 6.3 | 328,625 | 6.7 | 277,048 | 5.6 |
| **Traditional communities** |  |  |  |  |  |  |  |  |  |  |  |  |  |  |  |  |  |  |
| Not declared member | 2,045,188 | 99.8 | 2,653,002 | 99.7 | 2,527,924 | 99.6 | 2,366,163 | 99.4 | 2,563,330 | 98.1 | 1,752,752 | 97.6 | 4,642,105 | 98.2 | 4,845,033 | 98.4 | 4,880,394 | 98.6 |
| Declared member | 4,929 | 0.2 | 8,319 | 0.3 | 9,876 | 0.4 | 14,387 | 0.6 | 49,708 | 1.9 | 43,197 | 2.4 | 86,115 | 1.8 | 80,548 | 1.6 | 68,163 | 1.4 |
| **Cash transfer recipient** |  |  |  |  |  |  |  |  |  |  |  |  |  |  |  |  |  |  |
| Not recipient | 764,358 | 37.3 | 803,979 | 30.2 | 848,359 | 33.4 | 842,559 | 35.4 | 763,191 | 29.2 | 483,815 | 26.9 | 1,303,261 | 27.6 | 1,622,228 | 32.9 | 1,457,286 | 29.5 |
| Recipient | 1,285,759 | 62.7 | 1,857,342 | 69.8 | 1,689,441 | 66.6 | 1,537,991 | 64.6 | 1,849,847 | 70.8 | 1,312,134 | 73.1 | 3,424,959 | 72.4 | 3,303,353 | 67.1 | 3,491,271 | 70.6 |
| **Region of residence** |  |  |  |  |  |  |  |  |  |  |  |  |  |  |  |  |  |  |
| North | 198,936 | 9.7 | 299,052 | 11.2 | 266,400 | 10.5 | 252,129 | 10.6 | 304,159 | 11.6 | 220,880 | 12.3 | 628,031 | 13.3 | 643,946 | 13.1 | 678,145 | 13.7 |
| Northeast | 877,691 | 42.8 | 1,153,141 | 43.3 | 1,093,301 | 43.1 | 995,620 | 41.8 | 1,087,879 | 41.6 | 711,583 | 39.6 | 1,893,465 | 40.1 | 1,914,285 | 38.9 | 1,970,064 | 39.8 |
| Central-West | 109,353 | 5.3 | 147,246 | 5.5 | 152,936 | 6.0 | 138,768 | 5.8 | 142,032 | 5.4 | 90,602 | 5.0 | 250,899 | 5.3 | 271,622 | 5.5 | 283,215 | 5.7 |
| Southeast | 529,049 | 25.8 | 666,874 | 25.1 | 632,301 | 24.9 | 629,443 | 26.4 | 739,421 | 28.3 | 544,322 | 30.3 | 1,391,305 | 29.4 | 1,455,896 | 29.6 | 1,411,954 | 28.5 |
| South | 335,088 | 16.3 | 395,008 | 14.8 | 392,862 | 15.5 | 364,590 | 15.3 | 339,547 | 13.0 | 228,562 | 12.7 | 564,520 | 11.9 | 639,832 | 13.0 | 605,179 | 12.2 |
| **Population size*** |  |  |  |  |  |  |  |  |  |  |  |  |  |  |  |  |  |  |
| Very small (≤20,000 ihn.) | 700,328 | 34.2 | 873,697 | 32.8 | 825,515 | 32.5 | 763,044 | 32.1 | 861,497 | 33.0 | 598,435 | 33.3 | 1,400,622 | 29.6 | 1,447,565 | 29.4 | 1,478,238 | 29.9 |
| Small (20,001-50,000 ihn.) | 526,907 | 25.7 | 697,653 | 26.2 | 654,989 | 25.8 | 602,351 | 25.3 | 671,472 | 25.7 | 452,807 | 25.2 | 1,170,708 | 24.8 | 1,204,895 | 24.5 | 1,248,882 | 25.2 |
| Medium (50,001-100,000 ihn.) | 276,557 | 13.5 | 365,421 | 13.7 | 349,854 | 13.8 | 325,468 | 13.7 | 363,868 | 13.9 | 247,103 | 13.8 | 668,939 | 14.2 | 684,812 | 13.9 | 686,134 | 13.9 |
| Large (>100,001 ihn.) | 546,325 | 26.7 | 724,550 | 27.2 | 707,442 | 27.9 | 689,687 | 29.0 | 716,201 | 27.4 | 497,604 | 27.7 | 1,487,951 | 31.5 | 1,588,309 | 32.3 | 1,535,303 | 31.0 |
| **Municipal HDI*** |  |  |  |  |  |  |  |  |  |  |  |  |  |  |  |  |  |  |
| Very low (0.000-0.499) | 11,457 | 0.6 | 17,028 | 0.6 | 16,542 | 0.7 | 15,354 | 0.6 | 19,414 | 0.7 | 15,821 | 0.9 | 39,552 | 0.8 | 39,538 | 0.8 | 44,192 | 0.9 |
| Low (0.500-0.599) | 505,469 | 24.7 | 683,547 | 25.7 | 633,544 | 25.0 | 570,096 | 24.0 | 672,573 | 25.7 | 458,386 | 25.5 | 1,160,529 | 24.5 | 1,155,506 | 23.5 | 1,216,770 | 24.6 |
| Medium (0.600-0.699) | 715,665 | 34.9 | 935,328 | 35.2 | 878,831 | 34.6 | 823,384 | 34.6 | 914,160 | 35.0 | 621,335 | 34.6 | 1,537,491 | 32.5 | 1,575,636 | 32.0 | 1,613,644 | 32.6 |
| High (0.700-0.799) | 756,406 | 36.9 | 956,102 | 35.9 | 943,632 | 37.2 | 899,839 | 37.8 | 914,026 | 35.0 | 633,658 | 35.3 | 1,763,263 | 37.3 | 1,874,083 | 38.1 | 1,843,645 | 37.3 |
| Very high (0.800-1.000) | 61,120 | 3.0 | 69,316 | 2.6 | 65,251 | 2.6 | 71,877 | 3.0 | 92,865 | 3.6 | 66,749 | 3.7 | 227,385 | 4.8 | 280,818 | 5.7 | 230,306 | 4.7 |
| **Gini index*** |  |  |  |  |  |  |  |  |  |  |  |  |  |  |  |  |  |  |
| Q1 lowest | 498,364 | 24.3 | 603,630 | 22.7 | 584,986 | 23.1 | 540,620 | 22.7 | 567,447 | 21.7 | 386,852 | 21.5 | 963,712 | 20.4 | 1,027,220 | 20.9 | 1,045,760 | 21.1 |
| Q2 | 494,931 | 24.1 | 621,209 | 23.3 | 586,709 | 23.1 | 553,873 | 23.3 | 600,338 | 23.0 | 414,544 | 23.1 | 1,054,497 | 22.3 | 1,100,939 | 22.4 | 1,089,124 | 22.0 |
| Q3 | 382,002 | 18.6 | 500,377 | 18.8 | 468,684 | 18.5 | 444,812 | 18.7 | 480,714 | 18.4 | 329,432 | 18.3 | 852,300 | 18.0 | 879,162 | 17.9 | 869,390 | 17.6 |
| Q4 | 395,019 | 19.3 | 541,975 | 20.4 | 525,120 | 20.7 | 476,518 | 20.0 | 526,623 | 20.2 | 347,232 | 19.3 | 931,106 | 19.7 | 964,506 | 19.6 | 977,844 | 19.8 |
| Q5 highest | 279,801 | 13.7 | 394,130 | 14.8 | 372,301 | 14.7 | 364,727 | 15.3 | 437,916 | 16.8 | 317,889 | 17.7 | 926,605 | 19.6 | 953,754 | 19.4 | 966,439 | 19.5 |

n: absolute frequency; %: relative frequency; HDI: human development index.

* Sociodemographic characteristics of the municipality of residence. 2010 Demographic Census, IBGE.
